# Comparison between Conventional Blind Embryo Transfer and Embryo Transfer Based on Previously Measured Uterine Length

**Published:** 2014-11-01

**Authors:** Nasrin Saharkhiz, Roshan Nikbakht, Saghar Salehpour

**Affiliations:** 1 Infertility and Reproductive Health Research Center (IRHRC), Shahid Beheshti University of Medical Sciences, Tehran, Iran; 2 Department of Infertility and IVF, Imam Khomeini Hospital, Jondishapour University of Medical Sciences, Ahwaz, Iran

**Keywords:** Ultrasound, Embryo Transfer, Uterine

## Abstract

**Background:**

Embryo transfer (ET) is one of the most important steps in assisted re- productive technology (ART) cycles and affected by many factors namely the depth of embryo deposition in uterus. In this study, the outcomes of intracytoplasmic sperm injec- tion (ICSI) cycles after blind embryo transfer and embryo transfer based on previously measured uterine length using vaginal ultrasound were compared.

**Materials and Methods:**

This prospective randomised clinical trial included one hun- dred and forty non-donor fresh embryo transfers during January 2010 to June 2011. In group I, ET was performed using conventional (blind) method at 5-6cm from the external os, and in group II, ET was done at a depth of 1-1.5 cm from the uterine fundus based on previously measured uterine length using vaginal sonography. Appropriate statistical analysis was performed using Student’s t test and Chi-square or Fisher’s exact test. The software that we used was PASW statistics version 18. A p value <0.05 was considered statistically significant.

**Results:**

Chemical pregnancy rate was 28.7% in group I and 42.1% in group II, while the difference was not statistically significant (p=0.105). Clinical pregnancy, ongoing preg- nancy and implantation rates for group I were 21.2%, 17.7%, and 12.8%, while for group II were 33.9%, 33.9%, and 22.1, respectively. In group I and group II, abortion rates were 34.7% and 0%, respectively, indicating a statistically significant difference (p<0.005). No ectopic pregnancy occurred in two groups.

**Conclusion:**

The use of uterine length measurement during treatment cycle in order to place embryos at depth of 1-1.5cm from fundus significantly increases clinical and ongo- ing pregnancy and implantation rates, while leads to a decrease in abortion rate (Registra- tion Number: IRCT2014032512494N1).

## Introduction

Embryo transfer (ET) is one of the most important steps in assisted reproductive technology (ART) cycles. The goal of ET is to deliver good quality embryos in early stages of development (zygote to blastocyst) to a uterus with suitable endometrium. It has been demonstrated that even with the transfer of high quality embryos, the success rate of the ART program is low and only 15- 20% of the transferred embryos will implant (1). It has been also estimated that up to 85% of the embryos transferred fail to implant (2).

There are multiple factors affecting the success of embryo transfer such as: embryo quality, uterine contractions, use of tenaculum, easy or difficult transfer, volume of transfer media, transfer technique, and depth of uterine transfer, while some of these factors are more important than others. One of the factors impacting the ART cycles’ outcome is the embryo transfer technique (3). Some clinicians believe that the impact of the transfer technique on the in vitro fertilization (IVF) results is as important as embryo quality, while any difficulty in ET may influence the implantation rate, significantly (4). One of the important aspects of the transfer technique is the depth of embryo deposition. If the deposition site is too deep, the chances of catheter touching the fundus and damaging the endometrium are increased. It has been demonstrated that touching the endometrium can stimulate junctional zone contractions (5) which may increase the chance of ectopic pregnancy.

In a study by Woolcutt et al. regarding "blind transfer", they have reported high rate of touching fundus or tubal ostia (6), and some other studies have indicated increased risk of ectopic pregnancy in cases of transfer close to the uterine fundus (7, 8). Therefore, the depth of the embryo deposition in the uterine cavity may influence the implantation success rates (9). Chun et al. showed a direct relation between implantation and pregnancy success rates with the length of the uterine cavity (10). The best site for ET to achieve higher pregnancy rates seems to be at a distance >10 mm and <20 mm from the fundus (11). There are several methods for ET. In the blind transfer method (with or without clinical touch), the embryo is planted 5-6 cm from the external os (12, 13), in the second method, the ultrasonography-guided embryo transfer is applied (2, 14), and in the third method, the embryo is transferred based on previously measured uterine length by a sound (metal or plastic) (15, 16).

There has been some controversy over the advantage of performing ultrasonography during ET. The positive impact of this procedure on the pregnancy rate has been reported in several studies (16, 17). In contrast, some other studies have reported no advantage in using ultrasonographic guidance (18-20). Since in many IVF centres in Iran, ET is performed blindly; therefore, in this study, we aimed to compare the outcome of conventional (blind) ET method with the ET method based on previously uterine length measurement using vaginal sonography.

## Materials and Methods

This was a prospective randomized clinical trial performed during January 2010 to June 2011. One hundred and forty women undergoing intracytoplasmic sperm injection (ICSI) during this period in Infertility and Reproductive Health Research Center (IRHRC), Shahid Beheshti University of Medical Sciences, Tehran, Iran, were enrolled in the study. Donor cycles and frozen ET were excluded. The study was approved by the Research Committee of the Infertility and Reproductive Research Centre of Shahid Beheshti University. A signed written consent was obtained from all participants.

The type of stimulatory cycle [agonist (Superfact; Hoechst, Frankfurt, Germany) or antagonist (Cetrotide, EMDSerono, Inc., Germany)] was selected based on the age of woman and other factors. Ovarian stimulation was done using recombinant follicle-stimulating hormone (r-FSH; Gonal-F; Serono Laboratories Ltd., Geneva, Switzerland) or purified FSH (Merional or Fostimon; IBSA, Switzerland), in single or combination formula.

When the leading follicles reached 17-18mm in diameter, human chorionic gonadotropin (hCG) 10,000 units (IBSA, Switzerland) was administered. The uterine length was measured, on the day of hCG administration, by recording the distance from the external os to the end of uterine cavity using vaginal sonography. Oocyte retrieval was performed 34-36 hours later, while the day of ET was determined by convenience. The patients were randomly divided into two groups. In group I, ET was performed using conventional (blind) method based on the sense and experience of the physician. In group II, the ET was performed based on previously measured uterine length using vaginal ultrasound and the embryo deposition was done at the depth of 1-1.5 cm from the top of uterine cavity. Furthermore, all ETs on even days were enrolled in group I, whereas all ETs on odd days were enrolled in group II.

### Procedure

All women were placed in the lithotomy position (with an empty bladder) and a sterile metal speculum was placed to expose the cervix. The cervical mucus was cleared using ringer solution, then the external os washed with media (Ham’s F-10 liquid, Sigma, Germany). In all cases, a Cook catheter (COOK Medical, USA) was used. First the outer catheter and then the inner catheter that was loaded with the embryos was placed. In group I, embryos were blindly deposited at the middle portion of the uterine cavity, approximately at 5-6 cm distance from the external os and based on physician experience.

In group II, embryos were deposited at 1-1.5 cm from the uterine fundus based on the previously measured uterine length without touching fundus and without ultrasound use during the procedure.

After slow withdrawal of the catheter and speculum, all women rested for an hour.

The difficulty of the ET was determined by the physician. When the catheter easily passing through the cervical canal was denoted easy, whereas any resistance to the insertion of the catheter, requiring a tenaculum, or future time-consuming manipulations were denoted difficult ET.

For supporting the luteal phase, all patients received Cyclogest vaginal suppository (400 mg BID) (Actover, Alpharma, England). Fourteen days after ovum retrieval, beta-hCG (β-hCG) level was measured. Ultrasound was performed three weeks later to determine clinical pregnancy and at 10-12 weeks of gestation, to determine ongoing pregnancy.

### Statistical analysis

Appropriate statistical analysis was performed using Student’s t test and Chi-square or Fisher’s exact test. The software that we used was PASW statistics version18. The power analysis of study was 80% and a p value <0.05 was considered statistically significant.

## Results

A total of 140 fresh and non-donor embryo transfers were performed. The conventional blind method was performed on eighty cases of group I and the previously measured uterine length method using vaginal ultrasound was performed on 60 cases of group II. The baseline and clinical characteristics of patients including age, duration and type of infertility, and etiology of infertility were compared between two groups (Table 1). No statistically significant differences were found between the two groups in other variables such as type of stimulation, type of gonadotropin used, number of retrieved oocytes, number of transferred embryos, endometrial thickness, uterine length, and easy or difficult transfer (Table 2), except for the day of transfer that in group II at 72 hours was later than group I at 48 hours after ovum pick up.

According to our results, although the chemical pregnancy rate was higher in group II (42.1 vs. 28.7%), the difference was not statistically significant (p=0.105). The clinical pregnancy, ongoing pregnancy and implantation rates were higher in group II (33.9 vs. 17.7% and 22.1 vs. 12.8%, respectively), indicating that the difference is statistically significant.

Abortion rate was higher in group I (34.7 vs. 0%). Except for the demise of one embryo in a twin pregnancy, no other abortion in the first trimester was recorded in group II. No ectopic pregnancy was detected in both groups (Table 3).

**Table 1:** The comparison of baseline and clinical characteristics between two groups


Patient	Frequency (%)		P value
	Group I N=80	Group II N=60	

**Age^a^ (Y)**	31.68 ± 0.69	31.4 ± 82	0.790^b^ (NS)
**Type of infertility**			0.122^c^ (NS)
Primary	61 (76.2%)	52 (86.7%)	
Secondary	19 (23.8%)	8(13.3%)	
**Cause of infertility**			0.950^d^ (NS)
Female factors	24 (30.8%)	16 (26.7%)	
Unovulation	10 (12.5%)	9(15%)	
Tubal factor	25 (31.2%)	17 (28.3%)	
Male factors	37 (47.4%)	31 (51.7%)	
Female and male	11 (14.1%)	9(15%)	
Unexplained	6 (7.7%)	4(6.6%)	


Signification at the 5 precent level. a; Mean ± standard deviation, b; T test, c; Chi-square analysis, d; Fisher exact test and NS; Non-significant.

**Table 2:** The comparison of the other studied variables between two groups


Patient	Frequency (%)		P value
	Group I N=80	Group II N=60	

**Treatment cycle**			0.283^a^ (NS)
Agonist	49(61.3%)	42(70%)	
Antagonist	31(38.7%)	18(30%)	
**Drug**			0.151^a^ (NS)
Single	29(36.3%)	29(48.3%)	
Combination	51(63.7%)	31(51.7%)	
**Transfer type**			0.130^b^ (NS)
Easy	72(90%)	58(96.7%)	
Hard	8 (10%)	2 (3.3%)	
**Uterine length^c^**	74.94 ± 1.33	74.97 ± 1.21	0.989 (NS)
**Stimulation days^d^**	8.43 ± 0.20	8.40 ± 0.24	0.923^c^ (NS)
**No. of oocytes^c^**	8.62 ± 0.57	9.73 ± 0.80	0.252 (NS)
**No. of embryos^d^**	5.14 ± 0.40	5.50 ± 0.45	0.555^c^ (NS)
**No. of ET^d^**	2.42 ± 0.08	2.45 ± 0.10	0.848^c^ (NS)
**End thickness**	8.58 ± 0.24	8.72 ± 0.31	0.73 (NS)
**Transfer time^d^ (Hour)**	63.60 ± 2.18	69.20 ± 1.98	0.060^c^ (NS)


Signification at the 5 precent level. NS; Non-significant, ET; Embryo transfer, a; Chi-square analysis, b; Fisher exact test, c; T test, and d; Mean ± standard deviation.

**Table 3:** The comparison of pregnancy results between two groups


Patient	Frequency (%)		P value
	Group I N=80	Group II N=60	

**Chemical pregnancy**	23 (28.7%)	24 (42.1%)	0.105^a^ (NS)
**Clinical pregnancy**	17 (21.2%)	19 (33.9%)	0.0135^a^*
**Ongoing pregnancy**	14 (17.7%)	19 (33.9%)	0.0131^a^*
**Abortion rate**	8 (34.7%)	0 (0%)	0.005^a^*
**Implantation rate^b^(%)**	12.86 ± 2.71	22.12 ± 4.57	0.086^c^*


NS; Non-significant, ET; Embryo transfer, a; Chi-square analysis, b; Mean ± standard deviation, c; T test and *; significant(s).

## Discussion

Despite many advances in the practice of ART cycles, the implantation and clinical pregnancy success rates are low even for patients with many oocytes and good quality embryos (1, 2). ET technique and the depth of embryo deposition in uterus are important factors which could affect the results (3, 9, 10). The optimal depth of embryo deposition has been suggested to be 1-2 cm from fundus (11, 21, 22), but determination of this depth have been carried out by different methods that lead to controversial results.

In many IVF centres in Iran, the conventional (blind) method is used. Although ultrasonic-guided ET have been suggested to improve the outcomes in some studies (16, 17), some other studies have not confirmed this finding (18, 19). In a study by Lambers et al., the outcome of ART cycles using previously measured uterine length method did not differ from cycles using ultrasound-guided ET method (20). Full bladder during abdominal ultrasonography is difficult and painful for many patients and is time-consuming for the physicians, so it is better and easier to perform ET procedure by empty bladder. Our study indicated that embryo transfer based on previously measured uterine length method results in significantly higher pregnancy and implantation rates as compared with blind method.

Increased clinical and ongoing pregnancy and implantation rates in this method may be due to determination of exact and suitable depth of uterus for embryos placement that indicates the importance of the fundal site of uterus for better implantation. The length of uterus is a factor affecting the outcome of ART, whereas this measurement is different among women and changes during the drug stimulation and cycle to cycle (10, 23). Therefore, the blind method seems not to be a suitable method since ET is done in a certain depth of uterus for all patients.

A decrease in abortion rate in our study is another point to support the importance of better site determination in ET success.

One of the different points between two groups in our study was the time of ET, suggesting that in group II, the day 3 ET showed better result as compared to the day 2 ET, but was not significantly different. This difference may affect the results, but some studies have showed that day 2 ET and day 3 ET have had similar reproductive outcomes (24, 25).

## Conclusion

It seems that the detection of uterine length by ultrasound during the treatment cycle and performance of ET at the depth of 1-1.5 cm from fundus may improve the outcome of ART cycles as compared to a blind approach, while leads to a decreases in abortion rate.

## References

[1] Edwards RG (1995). Clinical approaches to increasing uterine receptivity during human implantation. Hum Reprod.

[2] Sallam HN, Sadek SS (2003). Ultrasound-guided embryo transfer: a meta-analysis of randomized controlled trials. Fertil Steril.

[3] Eytan O, Zaretsky U, Jaffa AJ, Elad D (2007). In vitro simulations of embryo transfer in a laboratory model of the uterus. J Biomech.

[4] Mansour RT, Aboulghar MA (2002). Optimizing the embryo transfer technique. Hum Reprod.

[5] Lesny P, Killick SR, Tetlow RL, Robinson J, Maguiness SD (1998). Embryo transfer--can we Learn anything new from the observation of junctional zone contractions?. Hum Reprod.

[6] Woolcott R, Stanger J (1997). Potentially important variables identified by transvaginal ultrasound-guided embryo transfer. Hum Reprod.

[7] Egbase PE, Al-Sharhan M, Grudzinskas JG (2000). Influence of position and length of uterus on implantation and clinical pregnancy rates in IVF and embryo transfer treatment cycles. Hum Reprod.

[8] Nazari A, Askari HA, Check JH, O’shaughnessy A (1993). Embryo transfer technique as a cause of ectopic pregnancy in in vitro fertilization. Fertil Steril.

[9] Abdel Salam Mahamed M (2010). The influence of the depth of embryo transfer into the uterine cavity on implantation rate. Middle East Fertil Soc J.

[10] Chun SS, Chung MJ, Chong GO, Park KS, Lee TH (2010). Relationship between the length of the uterine cavity and clinical pregnancy rates after in vitro fertilization or intracytoplasmic sperm injection. Fertil Steril.

[11] Tiras B, Polat M, Korucuoglu U, Zeyneloglu HB, Yarali H (2010). Impact of embryo replacement depth on in vitro fertilization and embryo transfer outcomes. Fertil Steril.

[12] Brinsden PR, Brinsden PR (2005). Oocyte recovery and embryo transfer technique for IVF. Textbook of in vitro fertilization and assisted reproduction.

[13] Van Weering HG, Schats R, McDonnell J, Vink JM, Vermeiden JP, Hompes PG (2002). The impact of the embryo transfer catheter on the pregnancy rate in IVF. Hum Reprod.

[14] Buckett WM (2003). A meta-analysis of ultransound-guided versus clinical touch embryo transfer. Fertil Steril.

[15] Knutzen V, Stratton CJ, Sher G, McNamee PI, Huang TT, Soto-Albors C (1992). Mock embryo transfer in early luteal phase, the cycle before in vitro fertilization and embryo transfer: a descriptive study. Fertil Steril.

[16] Foran EJ, Auyeung A, Williams MM, Odem RR (2000). Endometrial cavity length (ECL): evidence for cyclic growth throughout in vitro fertilization (IVF) stimulation cycles. Fertil Steril.

[17] Eskandar M, Abou-Setta AM, Almushait MA, El-Amin M, Mohmad SE (2008). Ultrasound guidance during embryo transfer: a prospective, single-operator, randomized, controlled trial. Fertil Steril.

[18] Drakeley AJ, Jorgensen A, Sklavounos J, Aust T, Gazvani R (2008). A randomized controlled clinical trial of 2295 ultrasound-guided embryo transfers. Hum Reprod.

[19] Garcia-Velasco JA, Isaza V, Martinez-Salazar J, Landazabal A, Requena A, Remohi J (2002). Transabdominal ultrasound-guided embryo transfer does not increase pregnancy rates in oocyte recipients. Fertil Steril.

[20] Lambers MJ, Dogan E, Kostelijk H, Lens JW, Schats R, Hompes PG (2006). Ultrasonographic-guided embryo transfer does not enhance pregnancy rates compared with embryo transfer based on previous uterine length measurement. Fertil Steril.

[21] Pacchiarotti A, Mohamed MA, Micara G, Tranquilli D, Linari A, Espinola SM (2007). The impact of the depth of embryo replacement on IVF outcome. J Assist Reprod Genet.

[22] Coroleu B, Barri PN, Carreras O, Martinez F, Parriego M, Hereter L (2002). The influence of the depth of embryo replacement into the uterine cavity on implantation rates after IVF: a controlled, ultrasound-guided study. Hum Reprod.

[23] Williams CD, Kaelberer DF, Pastore LM, Bateman BG (2004). Uterine cavity changes in patients undergoing ovarian stimulation for in vitro fertilization: implications for transcervical embryo transfer. Fertil Steril.

[24] Shahine LK, Milki AA, Westphal LM, Baker VL, Behr B, Lathi RB (2011). Day 2 versus day 3 embryo transfer in poor responders: a prospective randomized trial. Fertil Steril.

[25] Laverge H, De Sutter P, Van der Elst J, Dhont M (2001). A prospective, randomized study comparing day 2 and day 3 embryo transfer in human IVF. Hum Reprod.

